# Osseous metaplasia with mature bone formation of the thyroid gland: Three case reports

**DOI:** 10.3892/ol.2013.1475

**Published:** 2013-07-19

**Authors:** JI-SUN CHUN, RAN HONG, JUNG-A KIM

**Affiliations:** 1 Department of Plastic Surgery, College of Medicine, Chosun University, Gwangju, Donggu 501-759, South Korea; 2 Department of Pathology, College of Medicine, Chosun University, Gwangju, Donggu 501-759, South Korea; 3 Department of Complementary and Alternative Medicine, Chosun University Graduate School, Gwangju, Donggu 501-759, South Korea

**Keywords:** osseous metaplasia, bone, thyroid gland

## Abstract

Nodular hyperplasia (nodular or multinodular goiter) is the most common form of thyroid disease. These nodules may undergo secondary changes in the form of hemorrhages, calcification and cystic degeneration. However, osseous metaplasia with mature bone formation rarely occurs. The present study reports the cases of three female patients with thyroid nodules diagnosed as nodular hyperplasia with osseous metaplasia and mature bone formation. The patients underwent right lobectomy, near total thyroidectomy and total thyroidectomy, respectively. The clinical course of the patients following resection were unremarkable.

## Introduction

Nodular hyperplasia is the most common form of thyroid disease (1), while sporadic (nodular) goiter is the most common type to be observed in the United States. Although the pathogenesis of nodular goiter remains unknown (2), a mild dietary deficiency of iodine, a slight impairment of hormone synthesis, an increased level of iodine clearance by the kidneys, the presence of thyroid-stimulating immunoglobulins and an increased production of insulin-like growth factor have all been suggested as causes (3–5). The incidence of the disease in the general adult population is 3–5% clinically and 50% at autopsy (6,7). Histopathologically, a wide range of appearances may be observed in the form of secondary degenerative changes, including hemorrhages, calcification, hyalinization, fibrosis and cystic degeneration. Occasionally, osseous metaplasia may occur (1). However, mature bone formation in a thyroid nodule is a rare occurrence (8,9). The present study describes the cases of three female patients with thyroid nodules showing osseous metaplasia with mature bone formation. The Insititional Review Board of Chosun University Hospital waived the requirement for written informed consent due to the nature of the study.

## Case reports

### Case 1

**Figure 1:**
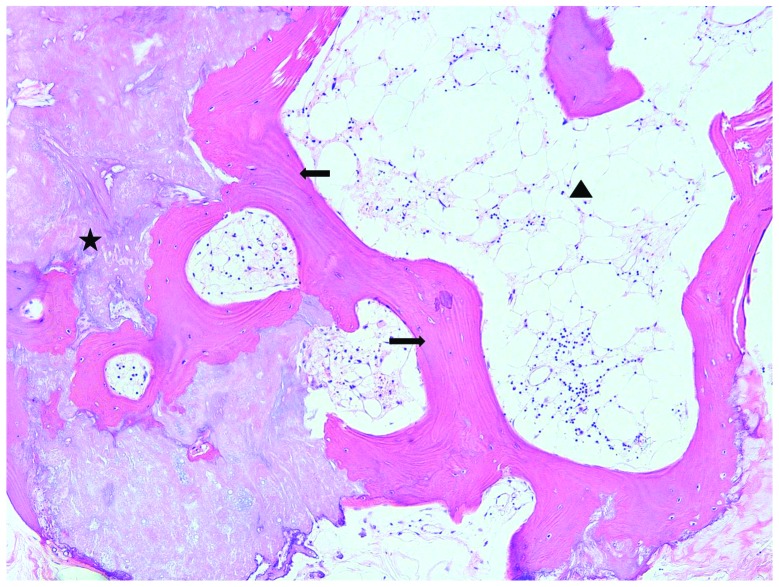
Case 1: A 41-year-old female. Small volume of osseous metaplasia (star), mature bony traculae (arrows) and fatty marrow (triangle; HE staining; magnification, ×200).HE, hematoxylin and eosin.

A 41-year-old female was examined in the Department of Endocrinology of Chosun University Hospital (Gwangju, South Korea) for thyroidal nodules due to the presence of thyroidal lesions that had been detected in a routine health examination three months prior to admittance to the hospital. The patient had no clinical symptoms, including endocrinological manifestations, compressive symptoms or palpable lumps. The serological thyroidal hormone levels were within the normal limits. Ultrasonography (US) of the thyroid revealed a 3-mm nodule in the right upper pole and a 10-mm nodule in the right lower lobe. The remaining thyroid gland was normal. A right lobectomy was performed. On gross examination, the cut surface revealed a 10×10-mm lesion, which was gray-yellow in color and had a bony-hard consistency. Microscopically, this lesion was a circumscribed mass composed of hyperplastic thyroidal follicles and mature bony trabeculae filled with mature fatty marrow (Fig. 1). The remaining thyroid gland presented with the appearance of nodular hyperplasia. The final diagnosis was nodular hyperplasia with mature bone formation.

### Case 2

**Figure 2:**
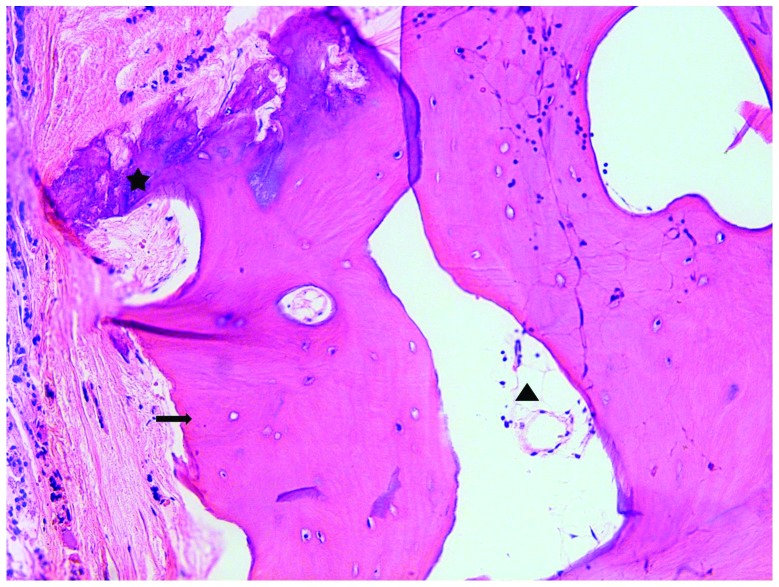
Case 2: A 49-year-old female. Osseous metaplasia (star), mature bony traculae (arrow) and fatty marrow (triangle; HE staining; magnification, ×100). HE, hematoxylin and eosin.

A 49-year-old female was admitted to the Surgery Clinic of Chosun University Hospital with a neck mass that had been present for several years. All the laboratory test results, including the thyroid hormone levels, were within the normal limits. The patient had not been previously treated with any form of irradiation and had no other disease present in the neck area. US examination of the thyroid revealed multiple thyroidal nodules with diameters measuring 40 mm in the left lobe, 16 mm in the isthmus, 7.5 mm in the right upper pole, 10 mm in the right mid-pole and 8.5 mm in the right lower lobe. A near total thyroidectomy was performed once the clinical diagnosis of a suspicious malignancy had been made. Grossly, multiple well-demarcated nodules were observed throughout the thyroid; a section from the right lobe demonstrated the presence of a mass with a hard consistency accompanied by calcification, which was microscopically shown to be a mature bone formation with fatty marrow and osseous metaplasia, as shown in Fig. 2. The remaining thyroid gland presented nodular hyperplasia showing dense hyalinization, fibrosis and cystic changes. The outcome of the patient following resection was unremarkable.

### Case 3

**Figure 3:**
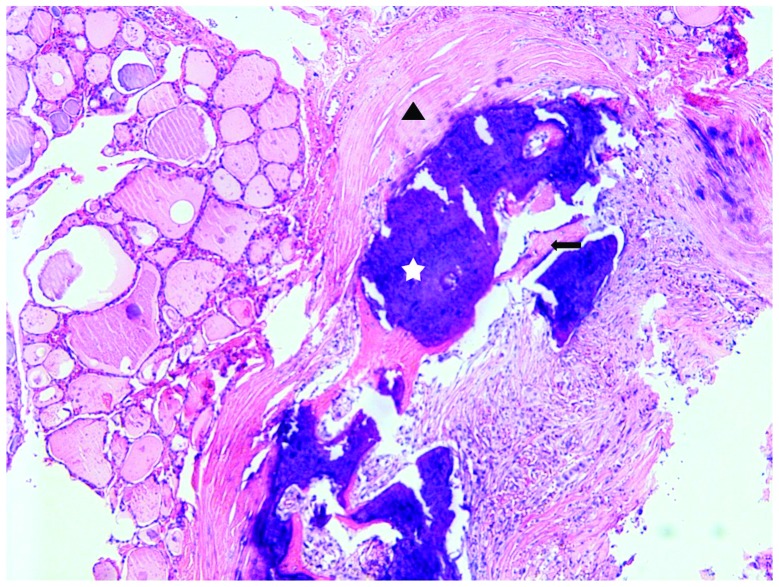
Case 3: A 72-year-old female. Extensive dystrophic calcification (star), small bony tissue (arrow) and thick fibrotic wall (triangle; HE staining; magnification, ×100). HE, hematoxylin and eosin.

The last case was of a 72-year-old female with a multinodular non-toxic goiter, which was detected by US of the thyroid. The laboratory test results, including the thyroid hormone levels, were within the normal limits. A total thyroidectomy was performed in the Department of ENT of Chosun University Hospital following the clinical diagnosis of a large-sized multinodular goiter. Histopathologically, there were multiple nodules showing secondary changes, including calcification, ossification and fibrosis in the resected specimen(Fig. 3).

## Discussion

Multinodular goiters, which are mainly caused by iodine deficiency, represent the most common form of thyroid disease in Europe and the United States, and degenerative changes are frequently observed (10,11). A rupture of the follicles leads to a granulomatous reaction to the colloid with the appearance of histiocytes and foreign body-type giant cells. Areas of fresh and old hemorrhages, coarse fibrous trabeculation and foci of calcification are common. Occasionally, osseous metaplasia is observed (1). Although dystrophic calcification may often be detected in a nodular goiter, maturation of this calcified tissue to mature bone is extremely rare (12).

The etiopathogenesis of osseous metaplasia remains unclear, although various theories have been proposed. A specific morphogenetic factor, bone morphogenetic factor (BMP), plays a significant role in bone formation, inducing local ossification and synthesizing a ground substance and collagen. However, the final step in bone formation depends on the presence of adequate concentrations of calcium and phosphate (12,13). The BMP family has at least 30 members, among which BMPs 1–7, which were initially isolated from demineralized bone matrix, are capable of inducing ectopic bone formation (14). Of these BMPs, BMP-1 is a type of metalloproteinase with conserved domains that is able to convert a variety of precursor proteins into mature or active forms that are involved in extracellular matrix formation (15–18). BMP-1 converts procollagen types I, II, III and VII into their mature forms and also mediates the C-terminal processing of the procollagen V homotrimer (16–18). A study by Basbug *et al*(19), which was conducted by the Tumor Research Center, showed that calcified thyroid tissue has a significantly higher expression of BMP-2 compared with that of normal thyroid tissue.

In conclusion, the present study reports the notable and unusual cases of multinodular goiter with osseous metaplasia and mature bone formation in three female patients.
